# Continuing Medical Education Speakers with High Evaluation Scores Use more Image-based Slides

**DOI:** 10.5811/westjem.2016.10.31484

**Published:** 2016-12-05

**Authors:** Ian Ferguson, Andrew W. Phillips, Michelle Lin

**Affiliations:** * Washington University School of Medicine, St. Louis, Missouri; † Stanford University, Department of Anesthesia, Division of Critical Care, Stanford, California; ‡ University of California, San Francisco, Department of Emergency Medicine, San Francisco, California

## Abstract

**Introduction:**

Although continuing medical education (CME) presentations are common across health professions, it is unknown whether slide design is independently associated with audience evaluations of the speaker. Based on the conceptual framework of Mayer’s theory of multimedia learning, this study aimed to determine whether image use and text density in presentation slides are associated with overall speaker evaluations.

**Methods:**

This retrospective analysis of six sequential CME conferences (two annual emergency medicine conferences over a three-year period) used a mixed linear regression model to assess whether post-conference speaker evaluations were associated with image fraction (percentage of image-based slides per presentation) and text density (number of words per slide).

**Results:**

A total of 105 unique lectures were given by 49 faculty members, and 1,222 evaluations (70.1% response rate) were available for analysis. On average, 47.4% (SD=25.36) of slides had at least one educationally-relevant image (image fraction). Image fraction significantly predicted overall higher evaluation scores [F(1, 100.676)=6.158, p=0.015] in the mixed linear regression model. The mean (SD) text density was 25.61 (8.14) words/slide but was not a significant predictor [F(1, 86.293)=0.55, p=0.815]. Of note, the individual speaker [χ^2^(1)=2.952, p=0.003] and speaker seniority [F(3, 59.713)=4.083, p=0.011] significantly predicted higher scores.

**Conclusion:**

This is the first published study to date assessing the linkage between slide design and CME speaker evaluations by an audience of practicing clinicians. The incorporation of images was associated with higher evaluation scores, in alignment with Mayer’s theory of multimedia learning. Contrary to this theory, however, text density showed no significant association, suggesting that these scores may be multifactorial. Professional development efforts should focus on teaching best practices in both slide design and presentation skills.

## INTRODUCTION

Slide-based presentations, such as Microsoft PowerPoint^TM^ and Apple Keynote^TM^, serve as a common format in continuing medical education (CME) conferences. Consequently, developing effective design principles for such multimedia presentations in health professions education is essential to optimize information delivery, attendee engagement, and adult learning.

Researchers have developed instructional design principles for multimedia learning based on cognitive psychology experiments on learning and instruction. Richard Mayer’s cognitive theory of multimedia learning particularly provides a conceptual framework to describe how learners process multimedia.^1^,^2^ According to Allan Paivio and modified by Mayer, individuals process materials into either a visual or auditory channel within their working memory, each having a finite capacity. This is known as the dual-coding theory.^3^ Adherence to design principles can optimize learning by balancing the cognitive load for each of these channels. Alley et al. refined Mayer’s design principles to the specific demands of scientific presentations.^4^ Key tenets include replacing text with visual representations of the evidence and reducing the number of words on a slide, while the presenter tells the story. Presentations using these principles have been shown to improve retention and transfer of new knowledge.^5^–^7^ Although such multimedia design principles are supported by established theoretical underpinnings and empirical learning experiments, relevant published studies primarily involved undergraduate and medical students in controlled laboratory or classroom learning environments.^5^–^8^ No research has yet determined whether these principles are generalizable to adult learners in the setting of CME conferences.

The purpose of this study was to assess the response to evidence-based multimedia design principles in CME conference presentations by an audience of practicing clinicians. Our primary endpoints were the association of image fraction (percentage of image-based slides per presentation) and text density (average number of words per slide) with speaker evaluation scores. We hypothesized that presentation slides with more image-based slides and fewer words would result in higher speaker evaluation scores compared to presentations that did not adhere to these design principles.

## METHODS

### Participants and Study Design

This retrospective study analyzed attendees’ evaluation scores of speakers from six sequential national emergency medicine (EM) CME conferences over a three-year period. More specifically, we extracted data from the High Risk Emergency Medicine (HREM) and Topics in Emergency Medicine (TEM) conferences for 2010, 2011, and 2012. The same institution’s academic emergency department hosted both of these conferences. A mixed linear regression model assessed whether speaker evaluations were associated with image fraction (percent of image-based slides per presentation) and text density (number of words per slide) as well as the speaker and his/her academic seniority. This study received exemption status by the institutional review board at the University of California, San Francisco.

### Data Collection

We collected three data elements for the six conferences, which included the following: conference attendee evaluations, slide content, and demographics for each speaker. Anonymized attendee evaluations of the speakers were provided to the study group by the conference planners. Each lecture was evaluated on a five-point Likert scale (1=poor, 5=excellent) in each of the domains of delivery, content, and practical value. We used the overall evaluation score, defined as the mean score across all three domains, as the primary outcome measure because multimedia, slide-based learning is a complex process that includes aspects of all three domains.

Each lecture was videotaped and archived by CMEDownload.com. A single study author viewed all of them and collected study data from each lecture (image fraction, text density, and total presentation time). In the pilot phase, the author team corroborated the data and collectively clarified definitions for image fraction and text density for the data collection protocol. Image fraction was defined as the number of image-based slides divided by the total number of slides in the presentation. An image-based slide was any slide with an educationally-relevant image contributing to its teaching point, such as a graph, table, diagram, or illustrative photo. Thus, we did not count non-educational images, such as animations, institutional logos, or personal photos, as “images.” For presentations repeated by the same speaker in a different conference or year, only the most recent presentation was included. We excluded presentations by one study author and one study collaborator.

Faculty demographics collected included gender and academic rank, defined as clinical instructor, assistant professor, associate professor, or full professor. This information was publically available on the conference brochure and/or an Internet search of their academic departments.

### Data Collection Protocol for Slide Content

The master data-collection form for slide content included the following elements: name of presenter, conference name, year, total presentation time, total number of slides (excluding the title, disclosure, objectives, and summary slides), time per slide, number of teaching points per slide, number of words per teaching point, and whether a slide included an educationally-relevant image (e.g. figure, chart, table, video). A “teaching point” was defined *a priori* as a discretely readable block of text, explicitly marked by bullets, numbers, or otherwise clearly separated. We excluded words embedded in figures, such as decision trees, tables, image captions, annotations, slide headers, citations, and journal article screenshots, from the final word count per slide.

### Statistical Analysis

We analyzed initial univariate tests for factors with theoretical association with overall speaker evaluation using independent t tests, univariate ANOVA, or Pearson’s *r* as appropriate, followed by a fixed multivariate regression for the naïve model, as is standard.^9^ The naïve model included the primary endpoints of image fraction (percentage of image-based slides, and calculated as a decimal value for analysis purposes) and text density (average words per slide).

This retrospective analysis contained a large number of lecturers who each gave a wide range of total presentations (range 1–8), and some speakers gave more than one presentation per conference. We therefore used a mixed linear regression for the final model, a common modeling method in the general education literature.^10^ (It is similar to a propensity score in that multiple factors are accounted for in a single variable.) In short, the mixed linear regression allows researchers to create a single variable that describes the variance for multiple related categorical factors, rather than create a new dummy variable for each of the categorical factors, thereby retaining statistical power.^9^

We entered all data initially into Excel 14.2.5, Microsoft Corporation, Seattle, Washington, and conducted all analyses using SPSS v21, IBM Corporation, Armonk, NY.

## RESULTS

Table 1 summarizes the data on conference lectures, evaluation response rates, and attendee clinical experience by conference and year. We analyzed a total of 105 unique presentations given by 49 faculty members from three High Risk EM (HREM) and three Topics in EM (TEM) CME conferences (2010–2012). From the video archive of 156 lectures, we included only 105 in this study; those excluded were repeat lectures, already included in the analysis, and lectures by two speakers who were involved in the design of this study.

The minimum and maximum number of lectures provided by a single presenter were one and eight, respectively, with a mean ± standard deviation (SD) of 2.14 ± 1.62 and median of two lectures. Speaker seniority was distributed by academic rank as follows: clinical instructor (n=2, 1.9%), assistant professor (n=44, 42.9%), associate professor (n=34, 32.4%), full professor (n=25, 23.8%). The mean evaluation score for all speakers was 4.50 ± 0.24 (SD) out of a maximum five points.

A total of 1,222 (70.1% response rate) evaluations were completed by conference attendees who collectively had 14.9 years (mean) of clinical experience. Clinical experience information was erroneously not captured in the 2010 TEM conference evaluation form.

Slide-set characteristics abstracted from the recorded lectures yielded an average image fraction of 0.47 ± 0.25, meaning that 47% of the slides in a presentation were image based. The mean text density (words per slide) was 25.61 ± 8.14.

### Univariate and Unadjusted Model Analyses

We performed initial univariate analyses to assess for potential factors in the model. Slide text density did not have a significant relationship with evaluations (r=−0.084, p=0.394). In contrast, image fraction was weakly associated with overall evaluation scores (r=0.197, p=0.044). We anticipated the possibility of a polynomial relationship between slide text density and image fraction with evaluation scores since too few and too many words or images may negatively impact evaluations. However, both scatter plots demonstrated linear relationships for the available data points.

The conference [F(5, 99)=3.49, p=0.006], speaker [F(48, 56)=3.30), p<0.001), and speaker seniority [F(3, 101)=5.89, p=0.001] were each associated with significant differences in mean evaluation scores in univariate tests. Total presentation time (r=0.009, p=0.928), time per slide (r=−0.072, p=0.464), and gender [t(103)=−0.963, p=0.338] were not significantly associated with mean evaluation scores.

An unadjusted model with slide image fraction and text density found a trend of image fraction predicting the mean evaluation [F(105)=3.489, p=0.065], while mean text density did not [F(105)=0.016, p=0.90]. Both primary endpoints were retained for the adjusted model because of their theoretical importance.

### Adjusted Model Analysis

We created a mixed linear regression model to account for violations of independence by presenters and conferences associated with the presentations that are required for a standard regression analysis. The final adjusted model included image fraction, slide text density, and speaker seniority as fixed effects. The speaker was represented as the random effects intercept. The total presentation time, conference, time per slide, and speaker’s gender did not significantly impact the model.

**Figure 1:**
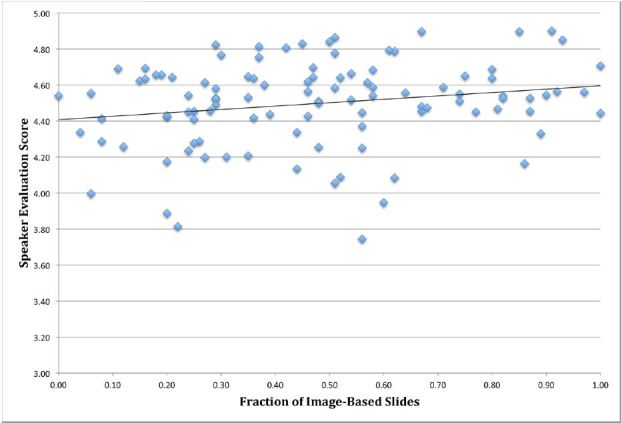
Unadjusted, univariate correlation between overall speaker evaluation scores (on a 5-point scale) and the fraction of image-based slides in their presentations.

The text density per slide did not significantly predict overall evaluation scores, [F(1, 86.293)=0.055, p=0.815], in the adjusted model. However, the image fraction significantly predicted overall evaluation scores [F(1, 100.676)=6.158, p=0.015] and had the greatest influence of any of the factors on predicting evaluation scores (b=0.277 on a 5-point Likert scale), as illustrated in Figure 1.

**Figure 2:**
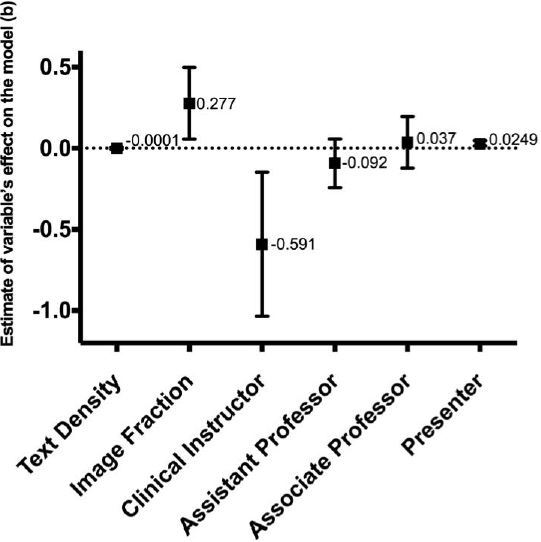
Interval plot showing the estimate of the variable’s effect on mixed linear regression model with 95% confidence intervals. Faculty seniority comparisons were made against full professor rank.

Seniority [F(3, 59.713)=4.083, p=0.011] and presenter [χ^2^(1)=2.952, p=0.003] also significantly predicted overall evaluation scores. (Presenter significance is given as χ^2^ because it was the random intercept in the mixed model.) The lowest-rank academic speakers (clinical instructor) received much lower evaluations, but this was in the context of only two speakers with this rank. Table 2 and Figure 2 present the estimates for all variables in the adjusted regression model.

## DISCUSSION

This is the first published study assessing the association between slide design and CME speaker evaluations by an audience of practicing clinicians. Higher evaluation scores were associated with presentations that had more image-based slides (image fraction) but, contrary to our hypothesis, not those with fewer words per slide (text density). Speaker seniority was also associated with higher scores. These three findings can be understood in the context of the existing literature and conceptual framework of Mayer’s theory of multimedia learning and the dual-coding theory.

Our primary study finding was that image fraction was associated with higher speaker evaluation scores. The mixed linear regression model demonstrated a b estimate of 0.277 for image fraction. Although this value seems relatively low, this is in the context of a 95% confidence interval that rises as high as 0.5. Furthermore, conference attendees limited their evaluation scores to a narrow range (3.5–5.0). The functional scale was only 1.5 points, of which 0.277 represents a potential 13% absolute change, which represents practical significance.

The association between the use of image-based slides and speaker scores aligns with the fundamental multimedia premise of Mayer’s theory. Several studies have demonstrated that students learn and retain knowledge better when viewing slides with written text plus graphics compared to written text alone.^5^–^7^,^11^ The incorporation of images, however, should be judiciously considered. Not all images are educationally valuable. Images should be used only if they are integral to the teaching point. Humorous icons or animations can distract from learning and violate the multimedia principle of coherence, which advocates for the elimination of extraneous written text, audio, or graphics.^1^ If included, images should be high resolution and large enough to be read by all audience members.^12^,^13^ Blurry and small images (figures or tables) may detract from the message and negatively impact learning.^14^ If needed, such images need to be redrawn, enlarged to the full screen size, or removed altogether.

In contrast to image use, text density was not associated with higher speaker evaluation scores, which is in opposition to Mayer’s theory and our hypothesis. Excess text would seem to violate the modality principle, which states that on-screen text should not be repeated aloud. This becomes distracting and adds unnecessarily redundant cognitive loads to both the visual and auditory channels in one’s working memory. Two explanations might explain why text density showed no association in our study. First, the speakers all incorporated a similar average number of words per slide (25.61 ± 8.14) within a narrow range. This may not have allowed adequate differentiation among the presentations. Second, the modality principle is not as applicable for presentations with many technical terms or symbols.^11^ CME conference topics generally present more complex concepts, compared to non-medical or more basic talks.

In addition to the use of image-based slides, evaluation scores were also associated with speaker seniority. Speaker qualities such as delivery, tone, and confidence may have contributed to these higher scores. Additionally, a speaker’s reputation and stature may also have influenced the evaluations.

Our findings argue for more professional development training in health professions education on evidence-based multimedia design principles for slide design, as well as speaking skills. The default templates for PowerPoint encourage poor design elements such as text-heavy bullet points. Instead, the slides should be thoughtfully designed with sound multimedia principles to accompany and supplement the speaker’s message. For CME conference planners and speakers, our study illustrates that slide design should not be an afterthought in planning a presentation because it can significantly affect learner satisfaction.

Subsequent research should focus on reproducing this study in CME conferences of other health professions specialties and larger audiences to ensure generalizability. Additionally one can compare the post-test knowledge from CME conference attendees whereby the same speaker gives his/her same presentation using a different slide-set at another CME conference.

## LIMITATIONS

There are several limitations, primarily revolving around the study methodology. The outcome measure was the mean speaker evaluation score. This was a score derived from the domains of delivery, content, and practical value. There was no specific domain for slide design. Many confounding variables likely affected the mean score, such as lecture environment and presentation topic, for which we did not account.

Additionally, the CME evaluation forms were not validated. As is common in many CME conferences, custom templates were used. In our study, all six conference events used a similar evaluation template. The response rate for the evaluation forms was 41–86% (mean approximately 70%). Although this may lead to nonresponse bias, this falls within the typical response rate range of 60–80%.^15^

Only one author viewed and recorded data from all of the 105 included presentations. Although this may have introduced human error and interpretive biases in the data collection process, a second author corroborated the text and image counts from sample slides in the pilot phase of finalizing the data collection protocol.

Our mixed linear regression study demonstrated an association between slide design and higher speaker evaluation scores, but this does not equate to causation. Theoretically, more skilled speakers may have been trained to use more image-based slides. Our study is the first to show at least an association between CME speaker scores and slide design.

Attendee evaluation scores on speaker quality do not necessarily equate to learning gains. The Institute of Medicine’s Committee on Planning a Continuing Health Care Professional Education Institute has advocated for validated evaluation forms with learning-oriented outcomes for continuing professional development. This committee identified that evaluations of the instructors are also important in the multifaceted research on professional development.^16^ Thus for our study, we felt that speaker evaluation scores were a reasonable initial outcome measure focusing on CME conferences. Furthermore, conference organizers can use them to assess speaker effectiveness and attractiveness for future engagement. Future studies should prospectively examine both short- and long-term knowledge retention using post-conference tests.

## CONCLUSION

Our study contributes to the growing literature by Mayer, Issa, and others studying and refining the effectiveness of multimedia design principles on slide-based presentations. Uniquely we focused on a novel learner population, the practicing clinician, in CME conference settings. Application of evidence-based design principles, such as incorporation of images into slides, and speaker seniority are associated with higher speaker evaluation scores. In contrast to design principles, however, text density showed no significant association with speaker evaluation scores. Formal professional development programs for health professions educators should focus on cultivating effective slide design and presentation skills.

## Figures and Tables

**Table 1:** Recorded conference lectures, evaluation response rates, and attendee clinical experience from the six included conferences. High Risk Emergency Medicine (HREM); Topics in Emergency Medicine (TEM) (^*^ - data were not collected for that conference year).

Variable	HREM 2010	HREM 2011	HREM 2012	TEM 2010	TEM 2011	TEM 2012	Total
Number of included lectures (total number of conference lectures)	15 (24)	13 (22)	22 (28)	9 (19)	17 (32)	29 (33)	105 (158)
Number of evaluations completed (% of total number of registered attendees)	266/380 (70%)	258/290 (84.2%)	149/245 (60.8%)	262/306 (85.6%)	204/320 (63.8%)	83/202 (41.1%)	1222/1743 (70.1%)
Attendee mean number of years in clinical practice	14	13	14	^*^	12	16	14.9

**Table 2:** Mixed linear regression model to predict speaker evaluations. Faculty seniority comparisons are against full professor rank.

Variable	Estimate of variable’s effect on the model (b)	Standard error	95% Confidence interval
Mean text density (words/slide)	−0.0001	0.004	[−0.008, 0.007]
Image fraction	0.277	0.112	[0.056, 0.498]
Faculty seniority			
Clinical instructor (n=2)	−0.591	0.221	[−1.035, −0.146]
Assistant professor (n=44)	−0.092	0.075	[−0.242, 0.057]
Associate professor (n=34)	0.037	0.079	[−0.122, 0.196]
Full professor (n=25)	n/a	n/a	n/a
Presenter	0.0249	0.0081	[0.0131, 0.0470]
